# Widely Targeted Metabolomics Analysis of Different Parts of *Salsola collina* Pall

**DOI:** 10.3390/molecules26041126

**Published:** 2021-02-20

**Authors:** Shipeng Li, Ye Chen, Ying Duan, Yinhui Zhao, Di Zhang, Liyan Zang, Huiyuan Ya

**Affiliations:** School of Food and Drug, College of Life Science, Luoyang Normal University, Jiqing Road 6, Luoyang 471934, China; lsp423000@163.com (S.L.); chenye8987013@163.com (Y.C.); duanying@mail.ustc.edu.cn (Y.D.); ZYH395810172@163.com (Y.Z.); Zhangdi2021@126.com (D.Z.); Z18438677751@163.com (L.Z.)

**Keywords:** *Salsola collina* Pall, widely targeted metabolomics, primary and secondary metabolites, differential metabolites

## Abstract

*Salsola collina* Pall has a long history of being used as a traditional medicine to treat hypertension, headache, insomnia, constipation and vertigo. However, only a few biologically active substances have been identified from *S. collina*. Here, the shoots and roots of *S. collina*, namely L-Sc and R-Sc, were studied. The primary and secondary metabolites were investigated using ultrahigh-performance liquid chromatography-electrospray ionization-tandem mass spectrometry (UPLC-ESI-MS/MS). A total of 637 putative metabolites were identified and these metabolites were mainly classified into ten different categories. Correlation analysis, hierarchical clustering analysis, principal component analysis and orthogonal partial least squares discriminant analysis of metabolites showed that the L-Sc samples could be clearly separated from the R-Sc samples. Differential accumulated metabolite analysis revealed that most of differential primary metabolites were significantly lower in the L-Sc than in the R-Sc. Conversely, the major differential secondary metabolites had higher levels in the L-Sc than in the R-Sc. Further analysis indicated that the flavonoids were the major putative antioxidant components and most of putative antioxidant components exhibited higher relative concentrations in the L-Sc than the R-Sc. These results improve our understanding of metabolite accumulation and provide a reference for the study of medicinal value in *S. collina*.

## 1. Introduction

*Salsola collina* Pall belongs to the family *Chenopodiaceae*, and is widespread in northeast, north, northwest, and southwest of China [1]. *S. collina* has a long history of use in folk medicine [2]. The whole herb of *S. collina* is commonly used as herbal drink to treat hypertension, headache, insomnia, constipation and vertigo in China and Korea [3,4,5]. In Russia, *S. collina* is one component of the biologically active food additive “Heparon”, which has a liver protective, anti-alcohol, anti-inflammatory, and mild cholagogue effect [6].

Modern medical research has revealed that *S. collina* as herbal medicine exhibits beneficial effects on the immune and gastrointestinal system, has anti-inflammatory, anti-bacterial, and anti-hypertensive effects, and prevents cholelithiasis, dry bowel and constipation [7]. The ethyl acetate extract of *S. collina* can improve gastric emptying by regulating gastrointestinal hormone excretion and the c-Kit/SCF signaling pathway, and promote intestinal propulsion by modulating plasma ghrelin, gastrin, the plasma ghrelin receptor, and vasoactive intestinal peptide receptor 2 in the duodenum and activating M-cholinergic receptor [8,9,10]. Meanwhile, the ethanol extract from *S. collina* exerts anti-oxidative and anti-cancer activities by regulating the cell cycle [11]. In addition, the aqueous extract from *S. collina* is an effective means for the prophylaxis of cholelithiasis [12].

Although pharmacological studies have proved that *S. collina* has high medicinal value, there are few studies on the identification of active substances. Only about 60 biological active ingredients have been detected from *S. collina,* including flavonoids, alkaloids, phenolic acids, organic acids, sterols, etc. [6,8,9,13,14,15,16,17,18,19,20]. The main compounds are flavonoids and phenolic acids. The 18 reported flavonoids mainly involve flavones, flavonols and isoflavones [6,14,17,18,19,20]. Twelve phenolic acids have been identified from *S. collina*, such as vanillic acid, ferulic acid, salicylic acid, etc. [8,9,17,18,19,20]. It is well-known that there are both chemical and pharmacological differences in different parts of herbs [21]. Nevertheless, all samples used in previous studies have been collected from the aboveground parts of *S. collina*. Hence, the research on the chemical composition of *S. collina* at different parts is lacking.

With the development of metabolomics, high-throughput methods such as ultrahigh-performance liquid chromatography-electrospray ionization-tandem mass spectrometry (UPLC-ESI-MS/MS) have been applied to analyze metabolite profiles and detect variations in the compositions of herbs [21,22,23,24,25,26,27,28]. In this study, we used a widely targeted metabolomics method to investigate the chemical composition in *S. collina*, and identify the differentially accumulated metabolites (DAMs) between the shoots and roots of *S. collina*. Our results shed light on the metabolic pathways underlying *S. collina* and provide a scientific basis for application of *S. collina*.

## 2. Materials and Methods

### 2.1. Plant Materials

**Figure 1:**
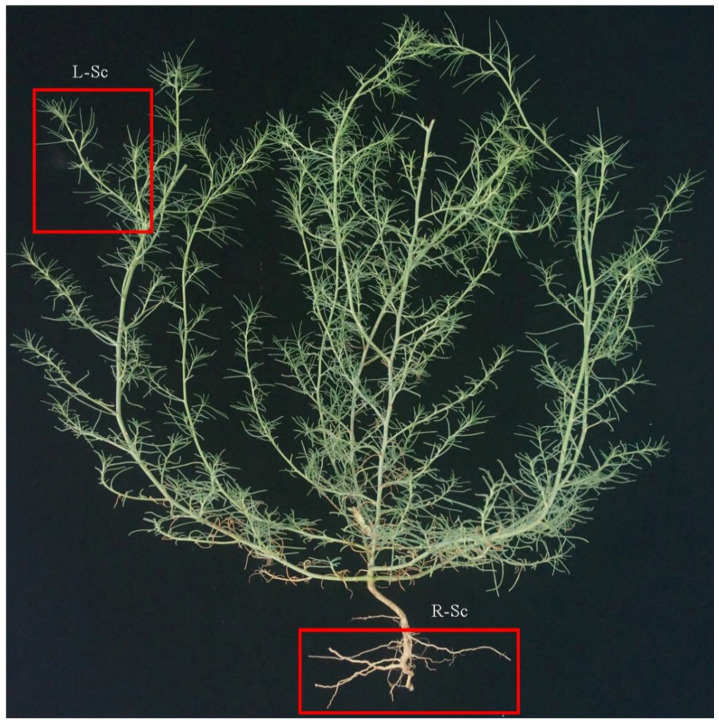
Phenotypes of *S. collina*.

All samples were collected from Luoyang, Henan Province, China, in the middle of July 2020. These samples were flash-frozen in liquid nitrogen containers, and stored at −80 °C for further analysis. The shoots and roots of *S. collina* were named L-Sc and R-Sc, respectively (Figure 1). For each sample, three biological replicates were independently analyzed. Five well-developed individuals were collected and combined for each repeat.

### 2.2. Sample Preparation and Extraction

The freeze-dried sample was crushed using a mixer mill (MM 400, Retsch) with a zirconia bead for 1.5 min at 30 Hz. A total of 100 mg powder was weighted and extracted overnight at 4 °C with 1.2 mL 70% aqueous methanol. Following centrifugation at 12,000 rpm for 10 min, the extracts were filtrated (SCAA-104, 0.22 μm pore size; ANPEL, Shanghai, China, http://www.anpel.com.cn/ (accessed on 2 February 2021)) before UPLC-MS/MS analysis.

### 2.3. UPLC Conditions

The sample extracts were analyzed using an UPLC-ESI-MS/MS system (UPLC, SHIMADZU Nexera X2, www.shimadzu.com.cn/ (accessed on 2 February 2021); MS, Applied Biosystems 4500 Q-TRAP, www.appliedbiosystems.com.cn/ (accessed on 2 February 2021)). The analytical conditions were as follows, UPLC: column, Agilent SB-C18 (1.8 µm, 2.1 mm × 100 mm); the mobile phase consisted of solvent A, pure water with 0.1% formic acid, and solvent B, acetonitrile with 0.1% formic acid. Sample measurements were performed with a gradient program that employed the starting conditions of 95% A, 5% B. Within 9 min, a linear gradient to 5% A, 95% B was programmed, and a composition of 5% A, 95% B was kept for 1 min. Subsequently, a composition of 95% A, 5.0% B was adjusted within 1.10 min and kept for 2.9 min. The column oven was set to 40 °C. The injection volume was 4 μL. The effluent was alternatively connected to an electrospray ionization (ESI)-triple quadrupole-linear ion trap (QTRAP)-MS

### 2.4. ESI-Q TRAP-MS/MS

Linear ion trap (LIT) and triple quadrupole (QQQ) scans were acquired on a triple quadrupole-linear ion trap mass spectrometer (Q-TRAP), AB4500 Q-TRAP-UPLC/MS/MS System, equipped with an ESI Turbo Ion-Spray interface, operating in positive and negative ion mode and controlled by Analyst 1.6.3 software (AB Sciex, Concord, Ontario, Canada). The ESI source operation parameters were as follows—ion source, turbo spray; source temperature 550 °C; ion spray voltage (IS) 5500 V (positive ion mode)/ −4500 V (negative ion mode); ion source gas I (GSI), gas 28 II (GSII), curtain gas (CUR) were set at 50, 60, and 25.0 psi, respectively; the collision gas (CAD) was high. Instrument tuning and mass calibration were performed with 10 and 100 μmol/L polypropylene glycol solutions in QQQ and LIT modes, respectively. QQQ scans were acquired as multiple reaction monitoring (MRM) experiments with collision gas (nitrogen) set to medium. The declustering potential (DP) and collision energy (CE) for individual MRM transitions were done with further DP and CE optimization. A specific set of MRM transitions were monitored for each period according to the metabolites eluted within this period [29].

### 2.5. Qualitative and Semi-Quantitative Analysis of Metabolites

The identification and structural analyses of the primary and secondary spectral data of the metabolites detected by mass spectrometry were based on the MWDB database (Metware Biotechnology Co., Ltd. Wuhan, China) and public databases, including MassBank (http://www.massbank.jp/ (accessed on 2 February 2021)), KNAPSAcK (http://kanaya.naist.jp/KNApSAcK/ (accessed on 2 February 2021)), HMDB (http://www.hmdb.ca/ (accessed on 2 February 2021)), MoToDB (http://www.ab.wur.nl/moto/ (accessed on 2 February 2021)), and ChemBank (http://chembank.med.harvard.edu/compounds (accessed on 2 February 2021)); PubChem (https://pubchemblog.ncbi.nlm.nih.gov/ (accessed on 2 February 2021)); NIST Chemistry Webbook (http://webbook.nist.gov/ (accessed on 2 February 2021)); and METLIN (http://metlin.scripps.edu/index.php (accessed on 2 February 2021)). Metabolomics data were processed using Analyst (Version 1.6.3, Sciex, Framingham, MA, USA).

Metabolite quantification was performed using MRM mode of QQQ mass spectrometry. In the MRM mode, the quadrupole filters the precursor ions of the target substance and excludes the ions corresponding to other molecular weights to eliminate interference. After obtaining the metabolite mass spectrometry data, peak area integration was performed using MultiQuant version 3.0.2 (AB SCIEX, Concord, ON, Canada). Finally, the chromatographic peak area was used to determine the relative metabolite contents.

### 2.6. Principal Component Analysis

Unsupervised principal component analysis (PCA) was performed by statistics function prcomp within R (www.r-project.org (accessed on 2 February 2021)). The data were unit variance scaled before unsupervised PCA.

### 2.7. Hierarchical Cluster Analysis and Pearson Correlation Coefficients

The hierarchical cluster analysis (HCA) results of samples and metabolites were presented as heatmaps with dendrograms, while Pearson correlation coefficients (PCC) between samples were calculated by the cor function in R (www.r-project.org (accessed on 2 February 2021)) and presented as only heatmaps. Both HCA and PCC were carried out by R package heatmap. For HCA, normalized signal intensities of metabolites (unit variance scaling) are visualized as a color spectrum.

### 2.8. Differential Metabolites Selected

Orthogonal partial least squares discriminant analysis (OPLS-DA) was performed on the identified metabolites. Differentially accumulated metabolites between groups were determined by fold change ≥ 2 or ≤0.5 and variable importance in project (VIP) ≥ 1. VIP values were extracted from OPLS-DA result, which also contain score plots and permutation plots, were generated using R package MetaboAnalystR (https://github.com/xia-lab/MetaboAnalystR (accessed on 2 February 2021)). The data were log transformed (log_2_) and mean centered before OPLS-DA. In order to avoid overfitting, a permutation test (200 permutations) was performed.

### 2.9. KEGG Annotation and Enrichment Analysis

Identified metabolites were annotated using the KEGG Compound database (http://www.kegg.jp/kegg/compound/ (accessed on 2 February 2021)), and annotated metabolites were then mapped to KEGG Pathway database (http://www.kegg.jp/kegg/pathway.html (accessed on 2 February 2021)). Pathways with significantly regulated metabolites mapped to were then fed into metabolite sets enrichment analysis (MSEA), and their significance was determined by the hypergeometric test’s *p*-values.

## 3. Results

### 3.1. Metabolic Profiling of S. collina Based on LC-MS/MS

**Figure 2:**
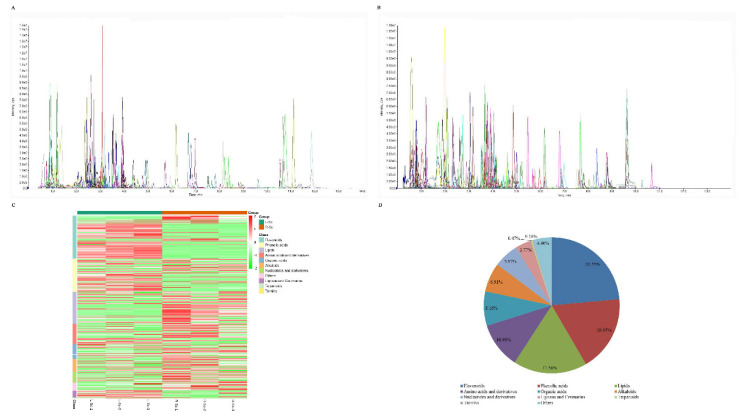
Qualitative and semi-quantitative analysis of metabolites in *S. collina*. Multipeak mass spectral chromatogram of metabolites acquired in negative ion mode (**A**) and positive ion mode (**B**). (**C**) Heatmap of the metabolites in the shoots and roots of *S. collina* (L-Sc and R-Sc, respectively). (**D**) Types and proportions of the identified metabolites from the L-Sc and the R-Sc.

To investigate the chemical composition in shoots and roots of *S. collina*, the primary and secondary metabolites were identified by LC-MS/MS analysis (Figure S1). The metabolites were quantitatively analyzed using software Analyst under the multiple reaction monitoring modes (Figure 2A,B). A total of 637 putative metabolites were detected and could be categorized into more than ten different classes, including 150 flavonoids, 115 phenolic acids, 112 lipids, 70 amino acids and derivatives, 52 organic acids, 44 alkaloids, 38 nucleotides and derivatives, 24 lignans and coumarins, three terpenoids, one tannin and 28 others (Figure 2C and Table S1). Flavonoids (23.55%), phenolic acids (18.05%), lipids (17.58%), amino acids and derivatives (10.99%) were the four main metabolites (Figure 2D). The flavonoids and lipids could be further categorized into nine and four classes, respectively (Table 1).

### 3.2. Multivariate Statistical Analysis

**Figure 3:**
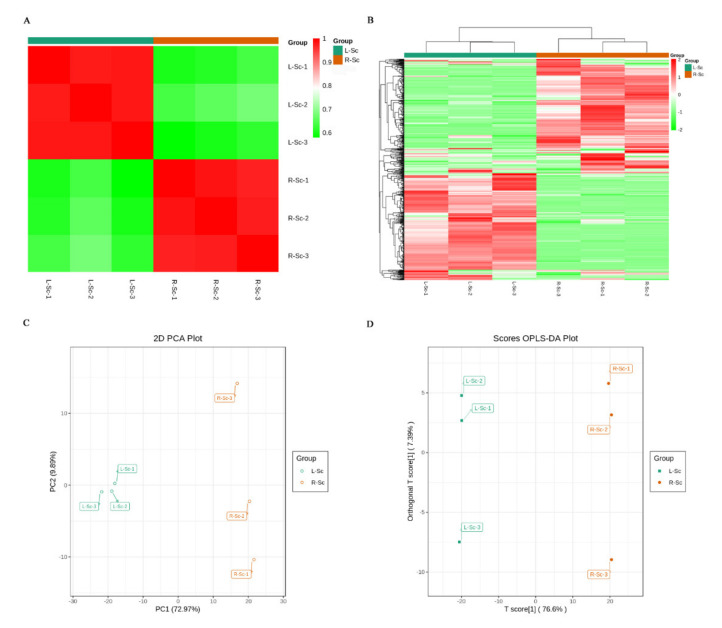
The identified metabolites analysis. (**A**) Pearson’s correlation coefficients among the L-Sc and R-Sc samples. (**B**) Cluster analysis of the identified metabolites from the L-Sc and the R-Sc. (**C**) Principal component analysis (PCA) of the L-Sc and R-Sc. (**D**) Orthogonal partial least squares discriminant analysis (OPLS-DA) model plot of the identified metabolites in the L-Sc and the R-Sc.

Multivariate statistics were used to assess further the identified metabolites in the L-Sc and the R-Sc. The correlation analysis showed that there was a significant correlation among biological replicates in both L-Sc and R-Sc (Figure 3A). Based on the hierarchical cluster analysis, the L-Sc samples and the R-Sc samples also could be clearly divided into two groups and the metabolites displayed different accumulation patterns between the two sets of samples (Figure 3B).

In order to further analyze the degree of variability in intergroup samples and intragroup samples, the metabolite profile of six samples was subjected to PCA (Figure 3C). Two principal components, PC1 and PC2, were extracted and explained 72.97% and 9.89% of the variability, respectively. In the PCA plot, the three biological replicates of L-Sc were concentrated in the middle of the left side of the plot, and the three biological replicates of R-Sc were distributed in the right side of the plot. The OPLS-DA mode was also used to screen the identified metabolites and evaluated the differential metabolites between L-Sc and R-Sc (Figure 3D). Like PCA, OPLS-DA also exhibited an obvious separation between L-Sc and R-Sc. We observed high predictability (Q2) and strong goodness of fit (R2X, R2Y) between HW and SW (Q2 = 0.986, R2X = 0.840, R2Y = 1.000), which demonstrated that these modes were stable and reliable and could be used to further identify the differentially accumulated metabolites (Figure S2). Taken together, these results suggested that there were significantly distinct metabolic profiles between L-Sc and R-Sc.

### 3.3. Differential Metabolites Between L-Sc and R-Sc

**Figure 4:**
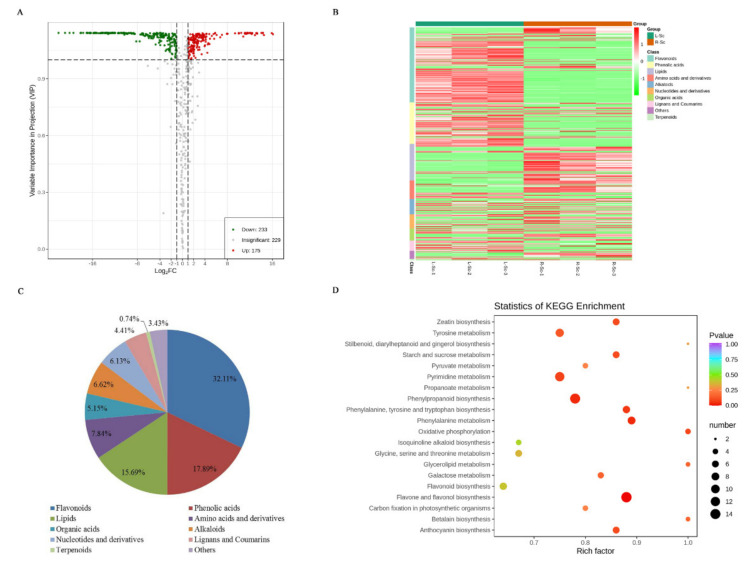
Differentially accumulated metabolites between L-Sc and R-Sc. (**A**) Volcano plot of the differential metabolites. (**B**) Heatmap of the differential metabolites. (**C**) Types and proportions of the differential metabolites. (**D**) Overview of KEGG pathway of the differential metabolites.

To identify differentially accumulated metabolites between L-Sc and R-Sc, a fold change ≥ 2 or a fold change ≤ 0.5 and variable importance in project (VIP) ≥ 1 were used as the screening criteria. A total of 408 DAMs were detected between L-Sc and R-Sc (Table S2). Of these, 233 putative metabolites were down-regulated and 175 putative metabolites were up-regulated in the R-Sc compared with the L-Sc (Figure 4A). The DAMs could be categorized into more than ten classes (Figure 4B), but the majority of DAMs were categorized into three classes, including flavonoids (32.11%), phenolic acids (17.89%) and lipids (15.69%; Figure 4C).

Table S3 shows the differential primary and secondary metabolites analysis. One hundred and thirty-three differential metabolites were primary metabolites, including 64 lipids, 32 amino acids and derivatives, 25 nucleotides and derivatives, nine saccharides and alcohols, and three vitamins. Compared with the L-Sc, most of differential primary metabolites, including 51 lipids, 22 amino acids and derivatives, 14 nucleotides and derivatives, and eight saccharides and alcohols, were up-regulated in R-Sc. About 30% of the differential primary metabolites, including 13 lipids, 11 nucleotides and derivatives, ten amino acids and derivatives, three vitamins, and one saccharide and alcohol, were down-regulated in the R-Sc. On the other hand, 275 differential metabolites belonged to secondary metabolites, including 131 flavonoids, 73 phenolic acids, 27 alkaloids, 21 organic acids, 18 lignans and coumarins, three terpenoids and two others. Unlike differential primary metabolites, only a few secondary metabolites were up-regulated in the R-Sc, including 27 flavonoids, 18 phenolic acids, 16 alkaloids, 12 organic acids, four lignans and coumarins, two terpenoids and one other. Most of them were down-regulated in the R-Sc, including 104 flavonoids, 55 phenolic acids, 14 lignans and coumarins, 11 alkaloids, nine organic acids, one terpenoid, and one other.

Furthermore, we mapped the metabolites from the L-Sc and the R-Sc to the KEGG database. A total of 231 metabolites, involving in 127 DAMs, were enriched in 80 pathways (Table S4). Among these pathways, metabolism was the largest category and only a few pathways were classified under genetic information processing and environmental information processing (Figure S3). The top enriched KEGG pathways were mainly involved in flavone and flavonol biosynthesis, biosynthesis of amino acids, phenylpropanoid biosynthesis, pyrimidine metabolism, and others (Figure 4D and Table S4).

### 3.4. Putative Antioxidant Components Analysis

**Figure 5:**
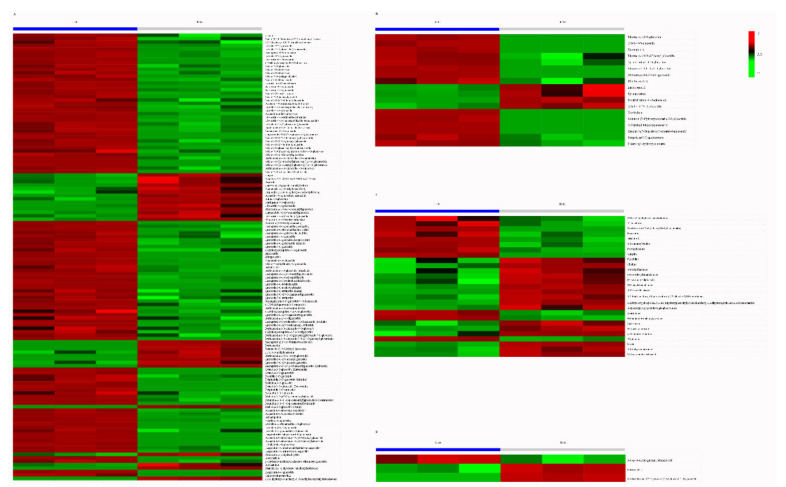
Heatmap of the differentially accumulated metabolites in potential antioxidant components between L-Sc and R-Sc. (**A**) Flavonoids, (**B**) lignans and coumarins, (**C**) alkaloids, and (**D**) terpenoids.

We further investigated the putative antioxidant components in the L-Sc and the R-Sc. Four types of putative antioxidant components were observed from the metabolite database, including flavonoids, lignans and coumarins, alkaloids, and terpenoids (Table 1). Most of putative antioxidant components were significantly different between L-Sc and R-Sc. Flavonesand flavonols were the major flavonoids, and most of flavonoids were found in higher relative concentrations in the L-Sc than in the R-Sc (Figure 5A and Table 2). Similarly, most of lignans and all the coumarins exhibited higher relative concentrations in the L-Sc than in the R-Sc (Figure 5B and Table 2). Conversely, more than half of alkaloids and terpenoids showed higher relative concentrations in the R-Sc than in the L-Sc (Figure 5C–D and Table 2).

## 4. Discussion

*S. collina* is well known for its medicinal benefits. However, only a few bioactive substances have been reported, and this has focused on flavonoids and phenolic compounds [6,8,9,10,14,17,20]. In our study, the primary and secondary metabolites of *S. collina* were analyzed widely, involving more than ten types of substances. Our results indicated that the main metabolites of *S. collina* were flavonoids, phenolic acids, lipids, amino acids and derivatives. Our results have greatly enriched the understanding of the chemical composition in *S. collina*.

The fresh *S. collina* is used as traditional medicinal materials or food additive, which can ease blood pressure, improve liver function and treat headache and vertigo [6,30]. The ethanol extract of *S. collina* has a good antihypertensive effect on senile or essential hypertension, and prolongs the hypnotic effect of pentobarbital sodium [2,7]. The recent research shows that the ethanol extract of *S. collina* also can promote gastric emptying and intestinal propulsion [8,9,10]. Flavonoids, alkaloids, lignins and coumarins, terpenes, polysaccharides, vitamins, etc., are recognized antihypertensive active ingredients. In this study, we identified a variety of hypotensive substances from *S. collina* (Table 1). Most of them belonged to flavonoids, alkaloids, and lignins and coumarins (Figure 2D). Therefore, we believe that the three substances are the main medicinal substances in *S. collina.*

In general, different parts of medicinal plants have unique medicinal values. The flower of Saffron is a valuable traditional Chinese medicine. Coniferin and crocin-2 are special components in stigmas and the content of flavonoids is high in tepals [31]. The root of *Platycodon grandiflorum* can be used as medicine. Compared with the stem, leaf and seed, the root contains more saponins [21]. Cortex moutan is made by drying the roots of peony, and its main roots have higher medicinal value than lateral roots [22]. Furthermore, the environment and growth age are also important factors affecting the medicinal value [24,32]. In this study, we found that the main putative antioxidant compounds showed higher relative concentrations in the shoots of *S. collina*, whereas a number of alkaloids and terpenoids were significantly higher in the roots of *S. collina* (Figure 5A and Table 2). Interestingly, the key of alkaloid, salsoline A, showed higher relative concentrations in the shoots of *S. collina* (Table S2). Therefore, we speculate that the shoots of *S. collina* have higher medicinal value.

## 5. Conclusions

In this study, we identified the chemical components of *S. collina* using a widely targeted metabolomics method. A total of 637 putative metabolites were detected in both the shoots and roots of *S. collina*. Flavonoids, alkaloids, lignans and coumarins were the main putative bioactive compounds, and most of them showed higher relative concentrations in the shoots of *S. collina*. The results improve our understanding of chemical components and medicinal value in *S. collina*.

## Figures and Tables

**Table 1:** Overview of the identified metabolites in *S. collina*.

Primary Classification	Secondary Classification	Number of Metabolites
Flavonoids	Flavones	63
	Flavonols	48
	Anthocyanins	13
	C-flavonoids	11
	Dihydroflavones	7
	Isoflavones	4
	Dihydroflavonols	2
	Dihydroisoflavone	1
	Flavanol	1
Phenolic acids	Phenolic acids	115
Lipids	Free fatty acids	39
	Lysophosphatidycholines	30
	Lysophosphatidyl ethanolamines	26
	Glycerol esters	17
Amino acids and derivatives	Amino acids and derivatives	70
Organic acids	Organic acids	52
Alkaloids	Alkaloids	26
	Phenolamines	11
	Plumeranes	7
Nucleotides and derivatives	Nucleotides and derivatives	38
Lignans and Coumarins	Lignans	17
	Coumarins	7
Terpenoids	Triterpene	1
	Triterpene Saponin	1
	Sesquiterpenoid	1
Tannins	Tannin	1
Others	Saccharides and Alcohols	17
	Vitamins	4
	Others	7

**Table 2:** Overview of the differentially accumulated metabolites in potential antioxidant components between L-Sc and R-Sc.

Primary Classification	Secondary Classification	L-Sc vs. R-Sc	
		Down	Up
Flavonoids	Flavones	41	14
	Flavonols	35	8
	Anthocyanins	11	1
	C-flavonoids	10	0
	Dihydroflavones	3	1
	Isoflavones	2	2
	Dihydroflavonol	1	0
	Flavanol	1	0
	Dihydroisoflavone	0	1
Lignans and Coumarins	Lignans	8	4
	Coumarins	6	0
Alkaloids	Alkaloids	8	10
	Phenolamines	2	3
	Plumeranes	1	3
Terpenoids	Triterpene	1	0
	Triterpene Saponin	0	1
	Sesquiterpenoid	0	1

## Data Availability

The data presented in this study are available in Supplementary Materials.

## Supplementary Materials

The supplementary materials are available online. Figure S1: Total ion chromatogram of the L-Sc and R-Sc under negative ion mode (A) and positive ion mode (B). Figure S2: OPLS-DA model validation diagram. Figure S3: KEGG pathway classification of the differential metabolites in the L-Sc vs. R-Sc dataset. Table S1: List and characteristics of the metabolites identified and quantified in *S.* collina. Table S2: List of differentially accumulated metabolites in the L-Sc vs. R-Sc dataset. Table S3: Statistics of differentially accumulated metabolites between L-Sc and R-Sc. Table S4: List of KEGG pathway in the L-Sc vs. R-Sc dataset.

## Sample Availability

Samples of the compounds are available from the authors.
