# Supplementary figures and images for: Widely Targeted Metabolomics Analysis of Different Parts of Salsola collina Pall
Source: Molecules. 2021 Feb 20;26(4):1126. doi: 10.3390/molecules26041126 (PMC7924207; doi:10.3390/molecules26041126)

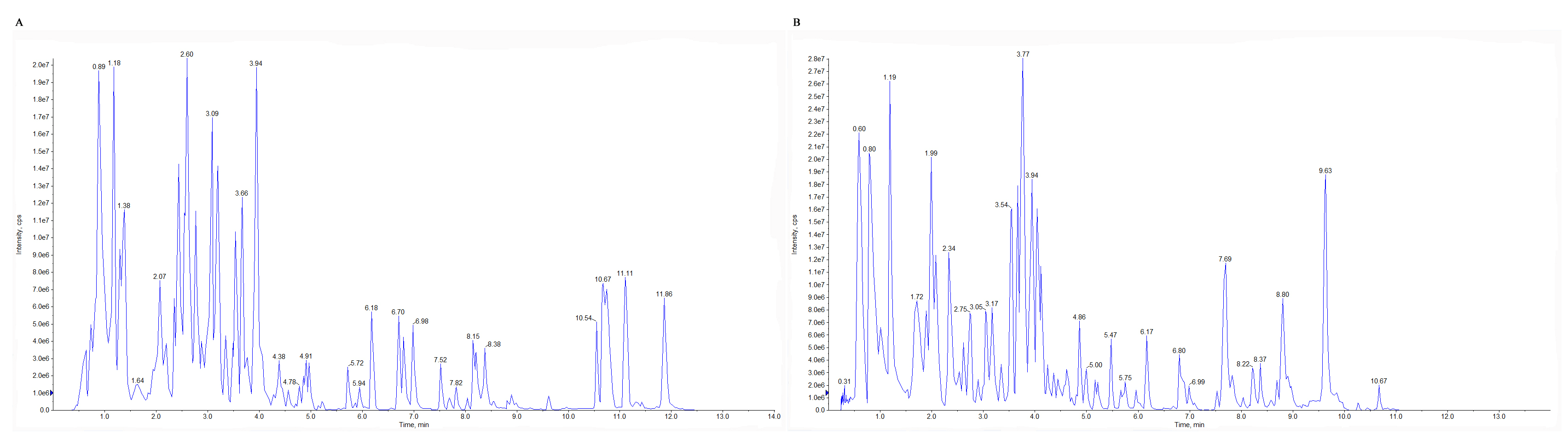

Supplement: Supplementary file 1 [file molecules-26-01126-s001.zip › molecules-1115436-supplementary-edited/Supplementary Files/Figure S1.tif]

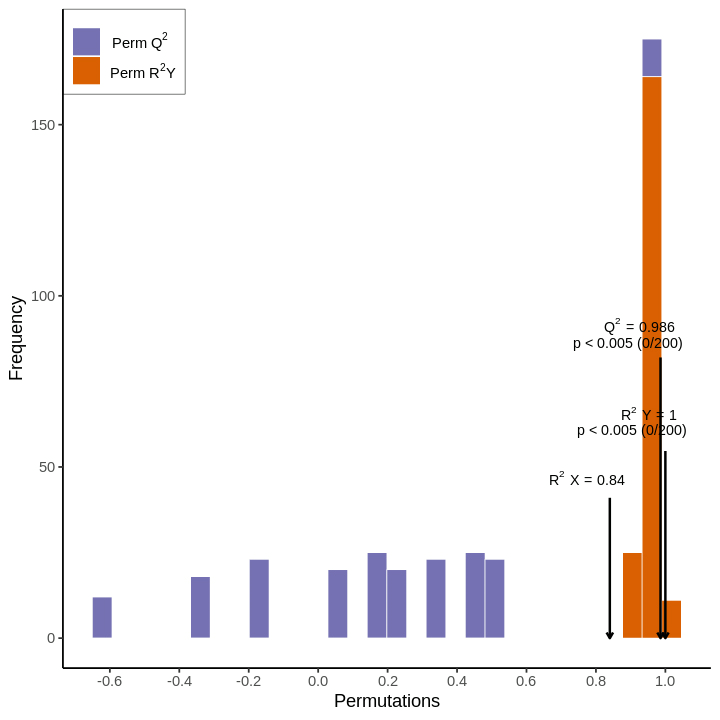

Supplement: Supplementary file 1 [file molecules-26-01126-s001.zip › molecules-1115436-supplementary-edited/Supplementary Files/Figure S2.tif]

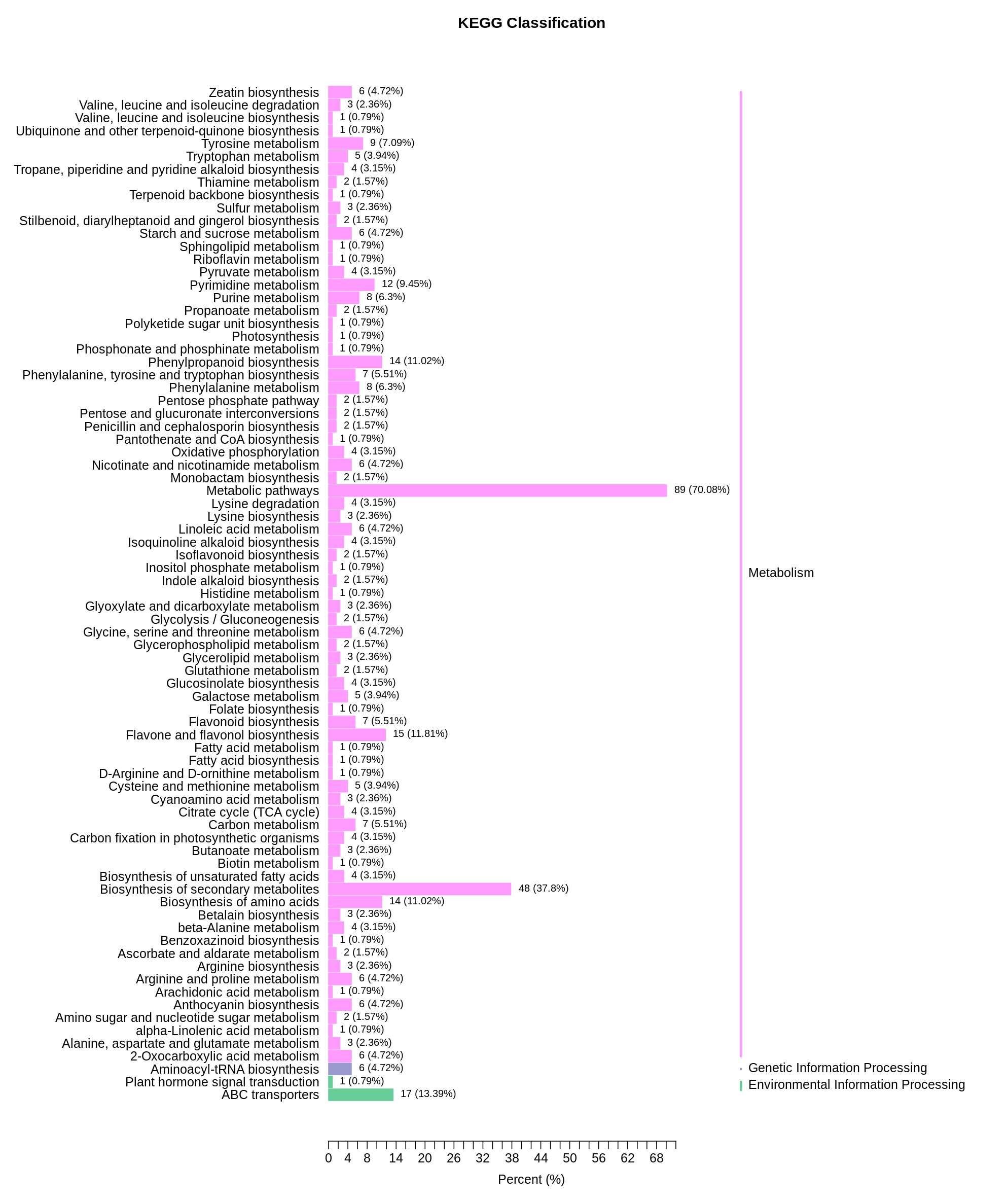

Supplement: Supplementary file 1 [file molecules-26-01126-s001.zip › molecules-1115436-supplementary-edited/Supplementary Files/Figure S3.tif]
